# miR-409-3p represses *Cited2* to refine neocortical layer V projection neuron identity

**DOI:** 10.3389/fnins.2022.931333

**Published:** 2022-09-29

**Authors:** Nikolaus R. Wagner, Ashis Sinha, Verl Siththanandan, Angelica M. Kowalchuk, Jessica L. MacDonald, Suzanne Tharin

**Affiliations:** ^1^Department of Biology, Program in Neuroscience, Syracuse University, Syracuse, NY, United States; ^2^Department of Neurosurgery, Stanford University Medical Center, Center for Academic Medicine, Palo Alto, CA, United States; ^3^Division of Neurosurgery, Palo Alto Veterans Affairs Health Care System, Palo Alto, CA, United States

**Keywords:** neocortical development, neuronal cell fate, callosal projection neuron, corticospinal motor neuron, microRNA

## Abstract

The evolutionary emergence of the corticospinal tract and corpus callosum are thought to underpin the expansion of complex motor and cognitive abilities in mammals. Molecular mechanisms regulating development of the neurons whose axons comprise these tracts, the corticospinal and callosal projection neurons, remain incompletely understood. Our previous work identified a genomic cluster of microRNAs (miRNAs), *Mirg*/12qF1, that is unique to placental mammals and specifically expressed by corticospinal neurons, and excluded from callosal projection neurons, during development. We found that one of these, miR-409-3p, can convert layer V callosal into corticospinal projection neurons, acting in part through repression of the transcriptional regulator *Lmo4*. Here we show that miR-409-3p also directly represses the transcriptional co-regulator *Cited2*, which is highly expressed by callosal projection neurons from the earliest stages of neurogenesis. *Cited2* is highly expressed by intermediate progenitor cells (IPCs) in the embryonic neocortex while *Mirg*, which encodes miR-409-3p, is excluded from these progenitors. miR-409-3p gain-of-function (GOF) in IPCs results in a phenocopy of established *Cited2* loss-of-function (LOF). At later developmental stages, both miR-409-3p GOF and *Cited2* LOF promote the expression of corticospinal at the expense of callosal projection neuron markers in layer V. Taken together, this work identifies previously undescribed roles for miR-409-3p in controlling IPC numbers and for *Cited2* in controlling callosal fate. Thus, miR-409-3p, possibly in cooperation with other *Mirg*/12qF1 miRNAs, represses *Cited2* as part of the multifaceted regulation of the refinement of neuronal cell fate within layer V, combining molecular regulation at multiple levels in both progenitors and post-mitotic neurons.

## Introduction

The cerebral cortex has undergone a remarkable expansion, including the evolution of distinct axonal tracts that have enabled the acquisition of complex motor and cognitive abilities [reviewed in Lui et al. (2011), Molnar et al. (2014), Fernandez et al. (2016)]. Callosal projection neurons and their associated axonal pathway in the corpus callosum are an evolutionary innovation thought to underpin the complex cognitive abilities of placental mammals. Callosal projection neurons are a broad population of interhemispheric projection neurons that extend an axon across the corpus callosum to connect the two cerebral hemispheres (Aboitiz and Montiel, 2003). The relative number of callosal projection neurons has expanded extensively throughout evolution of mammals, accounting for much of the neocortical thickness difference observed between macaque and human (Smart et al., 2002; Molnar et al., 2006; Fame et al., 2011). Callosal projection neurons are found throughout the layers of the neocortex; they are the predominant subtype of projection neuron in superficial layers (layers II/III), but about 20% of callosal projection neurons are found in deep layers, predominantly layer V. Deep layer callosal projection neurons are born at an earlier developmental stage than superficial layer callosal projection neurons and are molecularly distinct [reviewed in Fame et al. (2011)]. Although there are a small number of genes whose expression identify callosal projection neurons as a broad population, deep and superficial layer callosal projection neurons populations express distinct combinations of genes (Molyneaux et al., 2009, 2015; Klingler et al., 2019). Further, certain genes expressed by both, such as SATB2, appear to be regulated differently in deep layer callosal projection neurons than in superficial layer callosal projection neurons (Tashiro et al., 2011; Paolino et al., 2020). Deep layer callosal projection neurons, in fact, have been posited to be an evolutionarily older subpopulation of callosal projection neurons, perhaps forming the earliest interhemispheric connections.

The deep layer callosal projection neurons of layer V are generated concurrently with subcerebral projection neurons of layer V, predominantly corticospinal projection neurons [reviewed in Greig et al. (2013), Lodato and Arlotta (2015)]. The axons of the corticospinal projection neurons comprise the corticospinal tract, which is unique to mammals (Welniarz et al., 2017; Sahni et al., 2020). The full extension of the corticospinal tract to the lumbar segments that control walking is, like the corpus callosum, unique to placental mammals (Heffner and Masterton, 1975; Armand, 1982). The corticospinal tract is thought to underpin complex mammalian fine motor skills (Welniarz et al., 2017). Despite their divergent projections and functions, corticospinal motor neurons and layer V callosal projection neurons are generated at the same embryonic timepoint from the same progenitors (Greig et al., 2013; Lodato and Arlotta, 2015; Mancinelli and Lodato, 2018; Pinson and Huttner, 2021). The molecular mechanisms regulating the divergence of these distinct projection neuron populations are incompletely understood, however. Our recent work identified a genomic cluster of miRNAs (*Mirg*/12qF1) that is unique to placental mammals and specifically expressed by corticospinal neurons, and excluded from callosal projection neurons, during the molecular refinement of corticospinal and callosal fate (Diaz et al., 2020). One of these, miR-409-3p, has been shown to convert layer V callosal into corticospinal projection neurons, acting in part through the repression of the callosal-expressed transcriptional regulator *Lmo4* (Diaz et al., 2020). Our prior data suggest, however, that miR-409-3p represses additional callosal projection neuron control genes in order to specify corticospinal projection neurons.

One potential additional target of miR-409-3p repression is the transcriptional co-activator CBP/p300 Interacting Trans-activator 2 (*Cited2*). *Cited2* is highly expressed by callosal projection neurons, relative to corticospinal motor neurons, from the earliest stages of neurogenesis (Molyneaux et al., 2009), which is a reciprocal expression pattern to miR-409-3p. *Cited2* interacts genetically with *Lmo4* (Michell et al., 2010), and CITED2 and LMO4 function as opposing molecular controls over specific areal identity within superficial layer callosal projection neurons of the somatosensory and motor cortices, respectively (Fame et al., 2016). Expression of *Cited2* is evolutionarily conserved between macaque and mouse (Fame et al., 2017), including in the expanded subventricular zone (SVZ) and superficial layers of primates, and layer V callosal projection neurons. Our previous work demonstrated that CITED2 is necessary for the expansion of intermediate progenitor cells (IPCs) in the SVZ, and the resulting generation of superficial layer callosal projection neurons (Fame et al., 2016). Further, forebrain-specific *Cited2* conditional knockout (cKO) leads to behavioral deficits associated with human neurodevelopmental disorders (Wagner and MacDonald, 2021), highlighting the importance of CITED2 in cognitive function. Here, we investigate whether CITED2 is also necessary for the establishment of layer V callosal projection neurons, and whether it is repressed by members of the evolutionarily acquired *Mirg*/12qF1 miRNA cluster to refine the identity of layer V corticospinal projection neurons.

## Materials and methods

### miRNA target prediction

We searched for predicted miRNA targets using the search tools miRanda (Enright et al., 2003; John et al., 2004; Betel et al., 2008, 2010), Targetscan (Agarwal et al., 2015), DIANALAB (Maragkakis et al., 2009; Papadopoulos et al., 2009; Reczko et al., 2011), and miRDB (Wong and Wang, 2015; Liu and Wang, 2019).

### Luciferase assays

Luciferase reporter assays were performed using the Dual-Glo Luciferase Assay System (Promega), pmir-GLO based reporter constructs, and miRNA oligonucleotides (Horizon Discovery) according to manufacturer’s instructions, as previously described (Jin et al., 2013; Beard et al., 2016). Briefly, COS7 cells (10^4^/well) were seeded in a white 96-well plate. The following day, the media was replaced with transfection mixture, pmir-GLO reporter-miRNA oligo-DharmaFECT Duo (Dharmacon), and incubated overnight. Firefly and renilla luciferase reporter fluorescence was read using a Tecan Infinite M1000 (Stanford High-Throughput Bioscience Center Core Facility). The ratio of firefly to renilla fluorescence was calculated for each well, and averaged across triplicate wells. Match reporter vectors contained wild-type predicted miR-409-3p seed regions (CAACATT) with 30bp of flanking *Cited2* 3′ UTR on either side. Mismatch reporter vectors were identical to match reporters except that seed sequences were replaced by GGGGGGG. Additional negative controls using empty reporter vectors and scrambled control oligos were performed.

### Fluorescent activated cell sorting of intermediate progenitor cells

**FIGURE 1:**
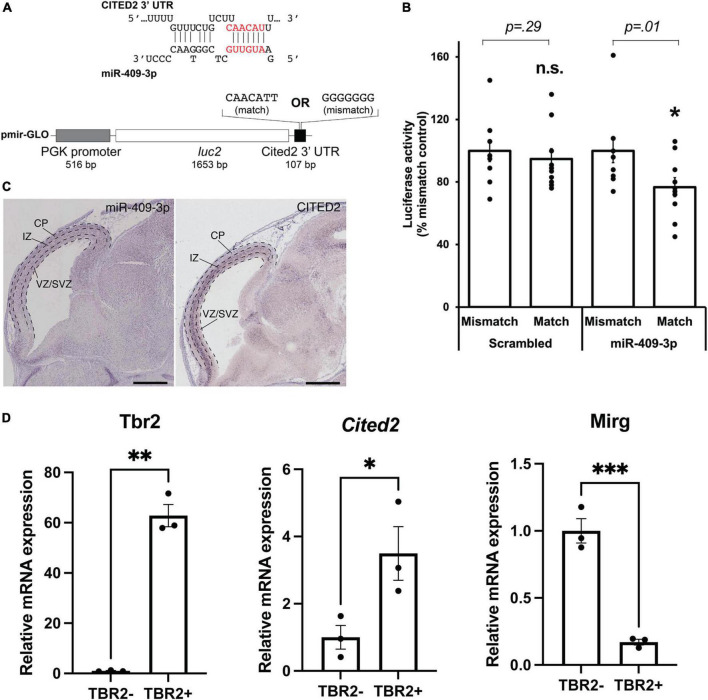
miR-409-3p represses the transcriptional regulator *Cited2*. **(A)** Sequence alignment demonstrating the predicted miR-409-3p target site in the Cited2 3′ UTR. Seed sequence base-pairing is in red. Luciferase reporter constructs were generated with the *Cited2* 3′ UTR fused to *luc2*, containing the wild-type predicted miR-409-3p target sequence (match). Mismatch reporter vectors were identical except that the target sequence was replaced by a sequence that would not be recognized by miR-409-3p (mismatch). **(B)** miR-409-3p oligonucleotides repress a Cited2 3′UTR luciferase reporter gene bearing wild-type, but not mismatch, miR-409-3p target sequences. Scrambled miRNA does not repress the Cited2 reporter. Error bars represent SEM. *p < 0.05 compared to mismatch control. **(C)** In the e14.5 dorsal telencephalon, miR-409-3p is enriched in the VZ and CP, and not in the upper SVZ and IZ, with minimal overlap with *Cited2*. Dashed lines indicate boundaries of the ventricular zone/sub-ventricular zone (VZ/SVZ), intermediate zone (IZ), and cortical plate (CP). Scale bars = 1 mm. Raw images from the Eurexpress database (Diez-Roux et al., 2011). **(D)** *Cited2* expression is enriched in IPCs at e15.5 by qPCR, while *Mirg*, which encodes miR-409-3p, is enriched in the TBR2- cells. Error bars represent SEM. Each dot depicts an independent biological replicate. *p < 0.05; ^**^p < 0.01 (Two-tailed *t*-test for *Cited2* and *Mirg*; Welch’s correction was performed for *Tbr2* due to unequal variance).

Sorting of IPCs for qPCR analysis was performed as previously described (Hrvatin et al., 2014; Baizabal et al., 2018). In brief, neocortical tissue from e15.5 embryos was dissected, dissociated, and pooled by genotype. Cells were fixed and resuspended in antibody buffer containing rabbit anti-TBR2 (Abcam cat# ab23345, RRID:AB_778267), followed by Alexa Fluor goat anti-rabbit 546 (1:750). Steps following dissociation were carried out with RNase-free reagents, treated with either 1:20 RNasin (recovery buffers), 1:40 RNasin (antibody buffers), or 1:100 RNasin (fixing solution and washing buffer). Cells were sorted at the Syracuse University Flow Core Facility with a FACS Aria II sorter (BD Biosciences) using an 85 μm nozzle and FACS Diva 8.0 software. Thresholds for 546 nm sorting gates were set using secondary only controls. Approximately 170,000–250,000 cells were collected for each population (TBR2 + and TBR2–) for each biological replicate. Successful sorting was validated with immunohistochemistry (Supplementary Figures 1A–A”) and qPCR (Figure 1D).

### mRNA quantitative-PCR

RNA was extracted from FAC-sorted samples using Recover All™ Total Nucleic Acid Isolation kit (Ambion). RNA quantity and quality were evaluated with Agilent RNA 6000 Pico chip with a 2100 Bioanalyzer (Agilent). cDNA was synthesized using qScript cDNA SuperMix (Quanta Biosciences). RT-qPCR was performed on a CFX Connect Real-Time System (Bio-Rad) using intron-spanning primer pairs to avoid genomic DNA amplification. Primers:

*Cited2:* forward 5′-GCTGTCCCTCTATGTGCTG-3′; reverse 5′-TGGTCTGCCATTTCCAGTC-3′

*Mirg:* forward 5′-TCGGCAGTACATACCAGGTG-3′; reverse 5′-ACTGATGGCTTCAGGTCAGG-3′ (Sanli et al., 2018)

*Tbr2:* forward 5′-CACCCAGAATCTCCTAACACTG-3′; reverse 5′-AGCCTCGGTTGGTATTTGTG-3′

*Housekeeping Reference Genes:*

*Gapdh*: forward 5′- GGC ATT GCT CTC AAT GAC AA -3′; reverse 5′- TGT GAG GGA GAT GCT CAG TG -3′

*S16*: forward 5′- CACTGCAAACGGGGAAATGG –3′; reverse 5′- TGA GA TGG ACT GTC GGA TGG –3′.

### Lentivirus vectors

Lentivirus vectors were modified from the pSicoR backbone (Ventura et al., 2004), a gift from Tyler Jacks (Addgene plasmid #11579). Expression of miRNA was under direction of the strong U6 promoter. miRNA inserts were either: miR-409-3p (GOF: GAATGTTGCTCGGTGAACCCCTTTTTT), or scrambled (control, CCTAAGGTTAAGTCGCCCTCGCTC CGAGGGCGACTTAACCTTAGGTTTTT). All miRNA inserts were cloned between *Hpa*I and *Xho*I sites. Expression of GFP was under direction of the CMV promoter. Lentivirus packaging was provided by System Biosciences (Palo Alto, CA). Titers of VSV-G pseudotyped viral particles were ∼10^7^ IFUS/mL.

### Cortical cultures

Primary cortical neuronal cultures were prepared as described previously (Diaz et al., 2020). Briefly, e14.5 CD-1 cortices were dissected and dissociated with gentle papain (Sigma) digestion. Cells were then plated on coverslips coated with poly-D-lysine (100 μg/ml, Sigma) alone or in combination with Laminin (20 μg/ml, Life Technologies). For miR409-3p GOF studies, cells were infected with lentivirus, and cultured on coverslips placed in 6-well plates for 2 days in growth media (50% DMEM, 50% neural basal media, supplemented with B27, BDNF, forskolin, insulin, transferrin, progesterone, putrescine, and sodium selenite). For *Cited2* knockdown experiments, 5 × 10^6^ cells were electroporated with 12.5 μg of *shScram* or *shCited2* plasmids (BTX ECM 830 Square Waveform Electroporation system, following the parameters: 700 V, one unipolar pulse at 100 μs pulse length in a 100 ms interval) and allowed to recover for 5 min. Cells were plated in Neurobasal media containing 10% fetal bovine serum, 1% GlutaMAX, and 1% penicillin-streptomycin (reagents from Thermo Fisher Scientific) at a density of 50,000 cells per coverslip in a 24-well plate. After 4 h, plating media was replaced with Neurobasal media containing 1% B27, 1% N2, 1% GlutaMAX, and 1% penicillin-streptomycin (reagents from Thermo Fisher Scientific). Half media changes were carried out every 2 days. Cells were maintained at 37°C, 5% CO2 until fixation. Knockdown of *Cited2* was analyzed by RT-qPCR, as out lined above, on duplicate wells to those analyzed by immunocytochemistry (Supplementary Figure 1B).

### Immunocytochemistry of cultured cells

Cells were fixed with 4% PFA in PBS. Coverslips were blocked with PBS containing 0.1% Triton-X100, 2% sheep serum, and 1% BSA, and cells were incubated with primary antibodies: anti-TBR2 (Abcam, rabbit polyclonal, cat# ab23345, RRID:AB_778267, 1:250), and/or anti-Tuj1 (Abcam, cat# ab7751, RRID:AB_306045, 1:200), anti-GFP (Life Technologies, cat# ab6662, RRID:AB_305635, 1:1000), anti-CTIP2 (Abcam, cat# ab18465, RRID:AB_2064130, 1:1000), anti-SATB2 (Abcam, cat# ab51502, RRID:AB_882455, 1:1000). Secondary antibodies: anti-rat (Pierce, CY3-conjugate, 1:1000), and/or anti-rabbit (Pierce, CY5-conjugate, 1:1000), and anti-GFP (Abcam, goat-FITC conjugated, 1:250).

For quantification, cells were imaged on a Zeiss AxioImager microscope. Transfected cells were counted in 16 randomly selected high-powered fields, blind to experimental condition, in 9 independent cultures. Statistical analyses were carried out in Microsoft Excel using paired two tailed *t*-tests. For quantification of effects of *Cited2* knockdown, 4 randomly selected fields per coverslip per condition were imaged using 10x objective on a Nikon Ni-U upright microscope, across 5 independent cultures. Statistical analyses were carried out in GraphPad Prism 8.0 (GraphPad Software) using one-way ANOVA followed by Tukey’s multiple comparisons test.

### *Cited2* conditional knockout mice

All animal experimental protocols were approved by the Syracuse University Institutional Animal Care and Use Committee and adhere to NIH ARRIVE guidelines. *Cited2* conditional floxed mice (Preis et al., 2006) were provided by Dr. Sally Dunwoodie, University of New South Wales, Australia. Emx1-Cre mice (Gorski et al., 2002) were obtained from The Jackson Laboratory (RRID:IMSR_JAX:005628). Breeding was performed as described previously (Wagner and MacDonald, 2021). Genotypes were assessed by PCR using the following primers: *Cited2* flox/flox, flox/wt, and wt/wt were determined using – *Cited2* Forward 5′-GTCTCAGCGTCTGCTCGTTT-3′; *Cited2* Reverse 5′-CTGCTGCTGTTGGTGATGAT-3′. Emx1 was distinguished from Emx1-Cre using – Emx1 WT Forward 5′-GAAGGGTTCCCACCATATCAACC-3′; Emx1 WT Reverse 5′-CATAGGGAAGGGGGACATGAGAG-3′; Emx1-Cre Reverse 5′-TGCGAACCTCATCACTCGTTGC-3′.

### Immunohistochemistry of tissue sections

Immunohistochemistry was performed as previously described (Fame et al., 2016). Briefly, E18.5 brains were post-fixed overnight in 4% PFA/PBS at 4°C, and sectioned on a VT1000S vibrating microtome (Leica Microsystems). Antigen retrieval was performed in 0.1M citric acid (pH = 6.0) for 10 min at 95–98°C. Sections were incubated in primary antibody at 4°C overnight (rat anti-CTIP2 (Abcam cat# ab18465, RRID:AB_2064130) and mouse anti-SATB2 [Abcam cat# ab51502, RRID:AB_882455)], and appropriate secondary antibodies were selected from the Molecular Probes Alexa series (Invitrogen, Carlsbad, CA). SATB2+, CTIP2+, and double positive cells were counted in layer V over a set distance on e18.5 coronal sections. Images were taken in the presumptive developing motor, somatosensory, and visual cortical areas based on the section’s alignment to the Atlas of the Developing Mouse Brain (Paxinos et al., 2007). All matching, imaging, and counting was performed by a researcher blinded to genotype. GraphPad Prism 8.0 (GraphPad Software) was used to carry out the statistical analyses; two-way ANOVA with Šídák’s multiple comparisons.

## Results

### miR-409-3p represses the transcriptional regulator *Cited2*

We have previously shown that miR-409-3p represses the callosal-expressed transcriptional activator *Lmo4* (Diaz et al., 2020). Our data suggest, however, that miR-409-3p represses additional callosal-expressed genes in order to specify corticospinal projection neurons. Bioinformatic analyses using the search tools miRanda (Enright et al., 2003; John et al., 2004; Betel et al., 2008, 2010), Targetscan (Lewis et al., 2005; Grimson et al., 2007; Friedman et al., 2009; Garcia et al., 2011; Agarwal et al., 2015), DIANALAB microT (Maragkakis et al., 2009; Papadopoulos et al., 2009; Reczko et al., 2011), and miRDB (Wong and Wang, 2015; Liu and Wang, 2019) predict that miR-409-3p represses a second callosal-expressed transcriptional regulator *Cited2*. Because *Cited2* and *Lmo4* are known to interact genetically during thymus development (Michell et al., 2010), and because *Cited2* and *Lmo4* cooperatively control callosal projection neuron areal identity, the predicted interaction of miR-409-3p with *Cited2* appeared to be highly biologically relevant. miR-409-3p is predicted to target a single site in the *Cited2* 3′ untranslated region (3′ UTR) (Figure 1A). To investigate whether miR-409-3p can use this site to repress gene expression, we performed luciferase reporter gene assays in COS7 cells, as previously described (Jin et al., 2013; Beard et al., 2016; Diaz et al., 2020). We used *Cited2* reporter vectors containing either wild-type or mutated (mismatch) miR-409-3p *Cited2* target sites and flanking 3′UTR sequences (Figure 1A). We found that miR-409-3p oligonucleotides significantly repress *Cited2* luciferase reporter gene expression with wild-type, but not mismatch, miR-409-3p target sequences (Figure 1B). Scrambled control miRNA oligonucleotides do not repress the *Cited2* luciferase reporter gene (Figure 1B).

In addition to its role in postmitotic callosal projection neuron development, *Cited2* regulates IPC function, specifically the number and proliferation of TBR2 + IPCs in the neocortex at e15.5, and thereby regulates the thickness of the mature superficial neocortex (Fame et al., 2016). *Cited2* and miR-409-3p appear to be expressed in non-overlapping cell populations in the e14.5 developing cortex by *in situ* hybridization (Diez-Roux et al., 2011; Figure 1C). *Cited2* is expressed in the SVZ, extending into the intermediate zone, as well as in a subset of cells in the cortical plate. miR-409-3p, on the other hand, is expressed in the VZ and cortical plate. To confirm that *Cited2* mRNA is enriched in e15.5 TBR2 + IPCs and that miR-409-3p is exclude from IPCs, we purified IPCs based on fluorescence activated cell sorting (FACS) of TBR2 + cells from e15.5 cortices. We confirmed the enrichment of TBR2 + IPCs *via* immunocytochemistry for TBR2 (Supplementary Figure 1A), and *via* RT-qPCR, demonstrating an approximate 55-fold enrichment in *Tbr2* in the TBR2 + cells versus the TBR2- cells (Figure 1D). Employing RT-qPCR, we confirmed that *Mirg*, the mRNA encoding miR-409-3p as part of a larger, polycistronic transcript and *Cited2* are reciprocally expressed in these cell populations, as would be predicted if miR-409-3p were repressing *Cited2* expression in TBR2- cells (Figure 1D). Collectively, the data suggest that miR-409-3p can repress expression of the IPC- and callosal projection neuron-expressed transcriptional regulator *Cited2*, potentially thereby regulating IPC cell number and proliferation, and subtype-specific cortical projection neuron development.

### miR-409-3p gain-of-function phenocopies *Cited2* loss-of-function, reducing the number of intermediate progenitor cells

**FIGURE 2:**
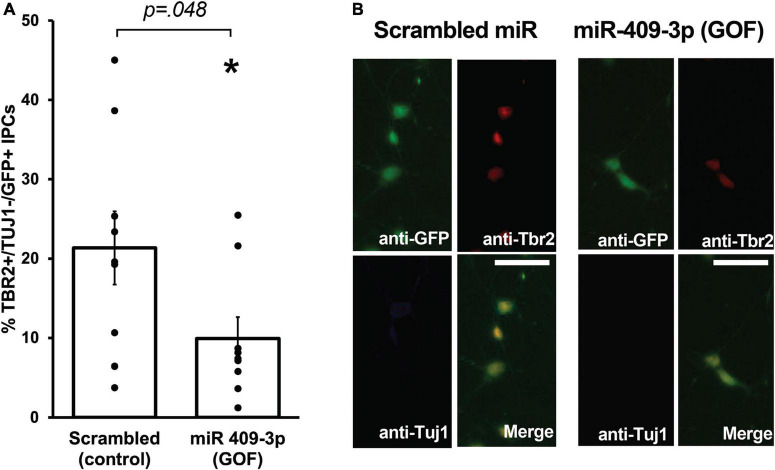
miR-409-3p represses intermediate progenitor cell proliferation in primary culture. **(A)** miR-409-3p overexpression gain-of-function (GOF) decreases the percent Tbr2 + /Tuj1-/GFP + IPCs compared to scrambled control in embryonic cortical cultures. **(B)** Representative fluorescence micrographs of embryonic cortical cultures illustrate a decrease in the percent Tbr2 + /Tuj1-/GFP + IPCs with miR-409-3p GOF. Scale bar, 50 μm. Each dot depicts an independent biological replicate. *p < 0.05 (Two-tailed *t*-test).

We showed previously that *Cited2* is required for the expansion of IPCs in the SVZ, and that *Cited2* LOF results in the generation of fewer TBR2 + IPCs at E15.5, likely as a result of reduced proliferation of IPCs rather than apoptotic cell death (Fame et al., 2016). To better understand the role of miR-409-3p in regulating neocortical development, we carried out miR-409-3p overexpression GOF experiments in primary cultures of embryonic cortical progenitors. Cultures of e14.5 cortical cells were transfected with lentiviral vectors expressing miR-409-3p and GFP or a control scrambled miRNA insert and GFP, and examined for cell type-specific protein expression by immunocytochemistry on day 2 in culture. To quantify IPCs and differentiated neurons within the GFP-positive transfected population, we analyzed expression of TBR2, a marker of IPCs, and Tuj1, a marker of differentiated neurons. Relative to scrambled control, miR-409-3p transfected cultures (GOF) display a significant decrease in TBR2 + /Tuj1- IPCs (Figures 2A,B), phenocopying the previously reported *in vivo Cited2* LOF (Fame et al., 2016), as would be predicted if miR-409-3p were repressing *Cited2* in cortical progenitors.

### *Cited2* loss-of-function phenocopies miR-409-3p gain-of-function, promoting callosal and repressing corticospinal projection neuron identity

**FIGURE 3:**
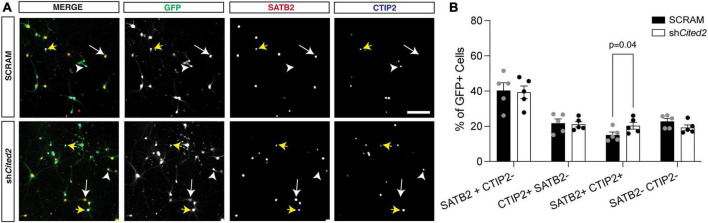
shRNA-mediated *Cited2* knockdown leads to an increase in neurons that co-express SATB2 and CTIP2 *in vitro*. **(A)** Representative micrographs of 7 DIV primary neuronal cultures electroporated with a control SCRAM or sh*Cited2.* Arrows indicate SATB2 + CTIP2- neurons (white arrow), CTIP2 + SATB2- neurons (white arrow head), and SATB2 + CTIP2 + co-expressing neurons (yellow arrows). **(B)** Quantification of transfected cells shows an increased percentage of SATB2 + CTIP2 + co-expressing neurons in sh*Cited2* vs SCRAM. *N* = 5. Two-way ANOVA with Šídák’s multiple comparisons test. Error bars denote SEM. Scale bar, 100 μm.

Our previous work demonstrated that overexpression GOF of miR-409-3p promotes corticospinal projection neuron identity in primary culture, in part *via* repression of *Lmo4* (Diaz et al., 2020). Because we suspected *Cited2* was also involved in this process, we assessed the effects of *Cited2* LOF on the percent corticospinal projection neurons relative to callosal projection neurons, hypothesizing that *Cited2* LOF would mimic phenotypes of miR-409-3p GOF. To evaluate effects of *Cited2* LOF on neuronal identity, e14.5 neocortical cultures were transfected with either a control scrambled shRNA (SCRAM) or an shRNA targeting *Cited2* (sh*Cited2*) and cultured for 7 days. Knockdown efficiency was assessed by RT-qPCR; there was an approximate 50% knockdown of *Cited2* across all cells in the culture (Supplementary Figure 1B), which aligns with the approximate 50% transfection efficiency. Cells were fixed and analyzed for neuronal subtype identity markers *via* immunohistochemistry. GFP + transfected cells were identified as SATB2 + CTIP2- (callosal projection neurons), SATB2- CTIP2 + (corticospinal motor neurons), or SATB2 + CTIP2 + (a subclass of developing corticospinal projection neurons) (Figure 3A). We find that *Cited2* LOF generates a significant increase in the percent of SATB2 + CTIP2 + co-labeled cells (Figure 3B) in a cell-autonomous manner, mirroring the overall increase in CTIP2 + cells observed with miR-409-3p GOF.

**FIGURE 4:**
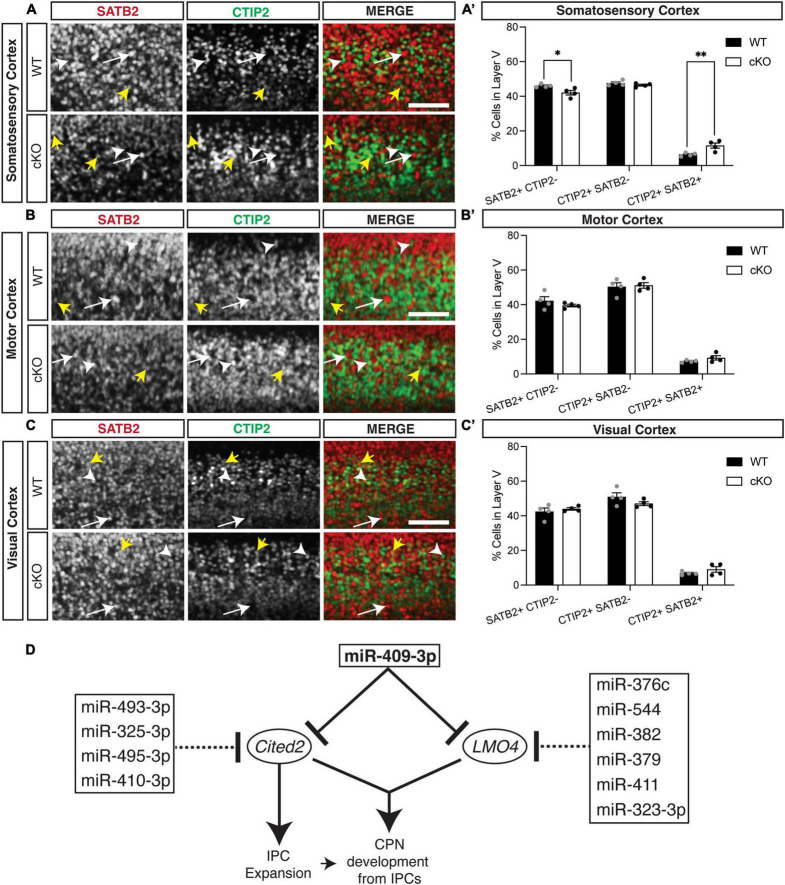
*Cited2* loss-of-function increases co-expressing SATB2 and CTIP2 cells at the expense of callosal projection neurons in layer V of the e18.5 developing somatosensory cortex. **(A,A′)** Conditional loss of *Cited2* generates fewer SATB2 + CTIP2- (white arrow) neurons and more SATB2 + CTIP2 + co-expressing neurons (yellow arrows) in layer V of the somatosensory region without altering the population of CTIP2 + SATB2- neurons (white arrow head). These differences were not observed in the developing motor region **(B,B′)** or visual region **(C,C′)**. *n* = 4. *p < 0.05; ^**^p < 0.01 (Two-way ANOVA with Šídák’s multiple comparisons test). Error bars denote SEM. Scale bars = 100 μm. **(D)** Schematic of the cooperative and multi-target repression of the Cited2-Lmo4 pathway for callosal projection neuron development by the corticospinal-expressed miRNAs of the *Mirg*/12qF1 cluster. miR-409-3p represses the interacting, callosal-expressed genes *Cited2* and *Lmo4* during cortical projection neuron development. Four corticospinal-enriched *Mirg*/12qF1 cluster miRNAs, in addition to miR-409-3p, are predicted to cooperatively repress Cited2. Six other corticospinal-enriched *Mirg*/12qF1 cluster miRNAs, in addition to miR-409-3p, are predicted to cooperatively repress Lmo4.

We also found previously that overexpression GOF of miR-409-3p (*via in utero* electroporation) promotes corticospinal projection neuron identity *in vivo* at e18.5 (Diaz et al., 2020). To determine if *Cited2* LOF phenocopies miR-409-3p GOF *in vivo*, we generated Emx1-Cre mediated forebrain-specific *Cited2* cKO mice, as previously described (Fame et al., 2016). We found that, at e18.5, *Cited2* LOF increases SATB2 + /CTIP2 + co-expressing cells, at the expense of deep-layer callosal projection neurons (Figure 4). This change is specific to the somatosensory cortex (Figures 4A,A′), where *Cited2* expression is maintained in the postmitotic neurons during neocortical arealization (Fame et al., 2016). Significant differences in the relative number of SATB2 + /CTIP2 + co-expressing cells are not observed in motor (Figures 4B,B′) or visual (Figures 4C,C′) cortices, where *Lmo4* is expressed. Taken together, our results suggest that miR-409-3p and *Cited2* exert opposing effects on corticospinal and callosal projection neuron development and support our model in which miR-409-3p represses *Cited2* to favor corticospinal over callosal fate.

## Discussion

Corticospinal and deep layer callosal projection neurons arise from the same pool of progenitors at the same time, but they go on to adopt completely different fates in support of completely different behaviors. The key, and broadly conserved, transcriptional regulators controlling cortical projection neuron fate, while critical, wholly account for neither the narrower evolutionary conservation of the corticospinal and callosal projections, nor for their own regulation of expression. We have previously shown that a cluster of miRNAs unique to placental mammals (*Mirg*/12qF1) is expressed by corticospinal projection neurons but not callosal projection neurons during their development, and that one of these miRNAs, miR-409-3p, can shape corticospinal over callosal projection neuron fate, acting in part *via* repression of the callosal-expressed transcriptional regulator LMO4. While our findings suggested additional targets for miR-409-3p and other miRNAs in the cluster, these were until now unknown. Here we show that miR-409-3p also targets the callosal-expressed, and *Lmo4*-interacting, transcriptional regulator *Cited2* in cortical projection neuron progenitors. Furthermore, we define previously undescribed roles for miR-409-3p in controlling IPC numbers and for *Cited2* in promoting callosal projection neuron fate.

The importance of non-coding RNAs, including miRNA, to neocortical expansion and evolution is emerging, with miRNA having been shown to play key roles in regulating multiple aspects of neurogenesis (Liu et al., 2021; Prodromidou and Matsas, 2021). Because *Cited2* is required for IPC expansion, the miR-409-3p/*Cited2* interaction led us to investigate a possible role for miR-409-3p in IPCs. *Cited2* is expressed by e15.5 IPCs while miR-409-3p is excluded from these progenitors (Figure 1D), as would be expected if miR-409-3p represses *Cited2*. miR-409-3p GOF decreases the percent TBR2 + IPCs in primary embryonic cortical cultures (Figures 2A,B), a phenocopy of *Cited2* LOF *in vivo* (Fame et al., 2016), further supporting a functional role for miR-409-3p repressing *Cited2*. However, *in vivo* analyses of miR-409-3p GOF will be necessary to determine whether there are temporal restrictions on this refinement.

miRNAs have previously been implicated in the production of cortical progenitors (Shibata et al., 2008, 2011; Gaughwin et al., 2011; Sun et al., 2011; Clovis et al., 2012; Dajas-Bailador et al., 2012; Nigro et al., 2012; Bian et al., 2013; Nowakowski et al., 2013; Zhao et al., 2013; Chen et al., 2014; Fei et al., 2014; Lv et al., 2014; Pollen et al., 2014; Pollock et al., 2014; Shin et al., 2014; Abdullah et al., 2016; Fededa et al., 2016; Zhang et al., 2016) and specifically in the proliferation of IPCs and expansion of layer 2/3 neuron generation (Tomasello et al., 2022), which are both disrupted in the *Cited2* cKO (Fame et al., 2016). However, no role for miR-409-3p in this process was known, and none of the previously described miRNAs have been shown to target *Cited2*. Our findings therefore provide a link between the role for miRNAs in IPC expansion and a transcriptional regulator known to control this process.

Beyond its role in IPC expansion, *Cited2* is required for the generation of superficial layer callosal projection neurons (Fame et al., 2016). However, its role in promoting deep layer callosal projection neuron development over corticospinal motor neuron development was previously unknown. In sensory cortex, our *in vivo* LOF findings demonstrate that *Cited2* promotes callosal projection neuron fate at the expense of corticospinal fate (Figures 4A,A′). Our *in vitro* data demonstrate that Cited2 LOF alters this fate in a cell-autonomous manner (Figure 3). These findings support a broad role for *Cited2* in promoting callosal projection neuron development, from IPC to deep layer to superficial layer callosal projection neurons. They also further support our previously published model that miRNA repression of transcription factors that promote callosal fate in corticospinal projection neurons contributed to the evolutionary emergence of layer V projections to the corpus callosum and corticospinal tract (Diaz et al., 2020).

The refinement of neuronal cell fate within layer V is multi-faceted, combining molecular regulation at multiple levels in both progenitors and post-mitotic neurons. Although SATB2 and CTIP2 are critical regulators of distinct neuronal lineages, and SATB2 represses CTIP2 (Alcamo et al., 2008; Britanova et al., 2008), they co-localize in a subset of developing projection neurons (Leone et al., 2008; Azim et al., 2009; Baranek et al., 2012; Harb et al., 2016). This co-localization has been posited to represent a developmental stage preceding fate refinement, with LMO4 essential for this delineation (Azim et al., 2009; Leone et al., 2015). However, double CTIP2/SATB2 expressing cells have also been posited to be a distinct neuronal subpopulation(s), which increase in abundance postnatally, particularly in the somatosensory cortex (Harb et al., 2016). We also noted the greatest abundance of CTIP2/SATB2 double expressing cells in the somatosensory cortex (Figure 4) in the *Cited2* cKO. However, as expression of *Cited2* becomes restricted to the somatosensory cortex, this likely represents the increased impact of *Cited2* loss-of-function on refinement of neuronal identity within the somatosensory cortex. To confirm the neuronal identity of the increased CTIP2/SATB2 double expressing cells within the *Cited2* cKO, however, it will be important to examine their axonal projections and assess additional neuronal cell type specific genes across additional timepoints.

Clustered miRNAs are known to cooperatively repress interacting genes within a pathway (Olena and Patton, 2010). *Lmo4* and *Cited2* appear to belong to such a pathway. The two genes have been shown to interact genetically during thymus development, including partial functional compensation for *Cited2* LOF by *Lmo4* in this system (Michell et al., 2010). In the developing cortex, *Cited2* and *Lmo4* appear to play region-specific roles in sculpting the areal identity of superficial layer callosal projection neurons in somatosensory and motor cortex, respectively (Fame et al., 2016). We have demonstrated that the *Mirg*/12qF1 miRNA miR-409-3p represses *Lmo4* (Diaz et al., 2020) and *Cited2* (Figure 1B). Bioinformatic analyses and work by other groups suggests that multiple other miRNAs from the *Mirg*/12qF1 cluster also target *Lmo4* or *Cited2* (Figure 4D).

Clustered miRNAs are also known to cooperatively repress the same genes (Olena and Patton, 2010). It was previously shown that two other *Mirg*-encoded 12qF1 miRNAs, miR-410 and miR-495, repress *Cited2* in cardiac myocytes during their development (Clark and Naya, 2015). Additionally, miR-495-3p has been shown to directly target *Cited2* and inhibit cell proliferation in cartilage endplate tissue (Zhang et al., 2021). Our bioinformatic analyses have identified and two additional *Mirg*/12qF1 miRNAs, miR-493-3p and miR-325-3p, predicted to cooperatively repress *Cited2*. There is thus convergent evidence supporting a role for the *Mirg*-encoded miRNAs of the 12qF1 cluster in repressing the transcriptional regulator *Cited2* during development. We propose a model whereby the clustered *Mirg*/12qF1 miRNAs cooperatively target the *Cited2-Lmo4* callosal development pathway for repression in corticospinal projection neurons, contributing not only to refinement of deep layer projection neuron fate, but also possibly to the evolution of the callosal and corticospinal projections in placental mammals.

## Data availability statement

The original contributions presented in this study are included in the article/Supplementary material, further inquiries can be directed to the corresponding authors.

## Ethics statement

The animal study was reviewed and approved by the Syracuse University Institutional Animal Care and Use Committee.

## Author contributions

JM, ST, NW, AS, and VS designed the research. NW, AS, AK, and VS performed the experiments. NW, AS, VS, AK, JM, and ST analyzed the data. JM and ST wrote the manuscript with contributions from all authors. All authors contributed to the article and approved the submitted version.
