# Supplementary material for: miR-409-3p represses Cited2 to refine neocortical layer V projection neuron identity
Source: Front Neurosci. 2022 Sep 29;16:931333. doi: 10.3389/fnins.2022.931333 (PMC9558290; doi:10.3389/fnins.2022.931333)
Supplement: Supplementary file 1 [file Image_1.PDF]

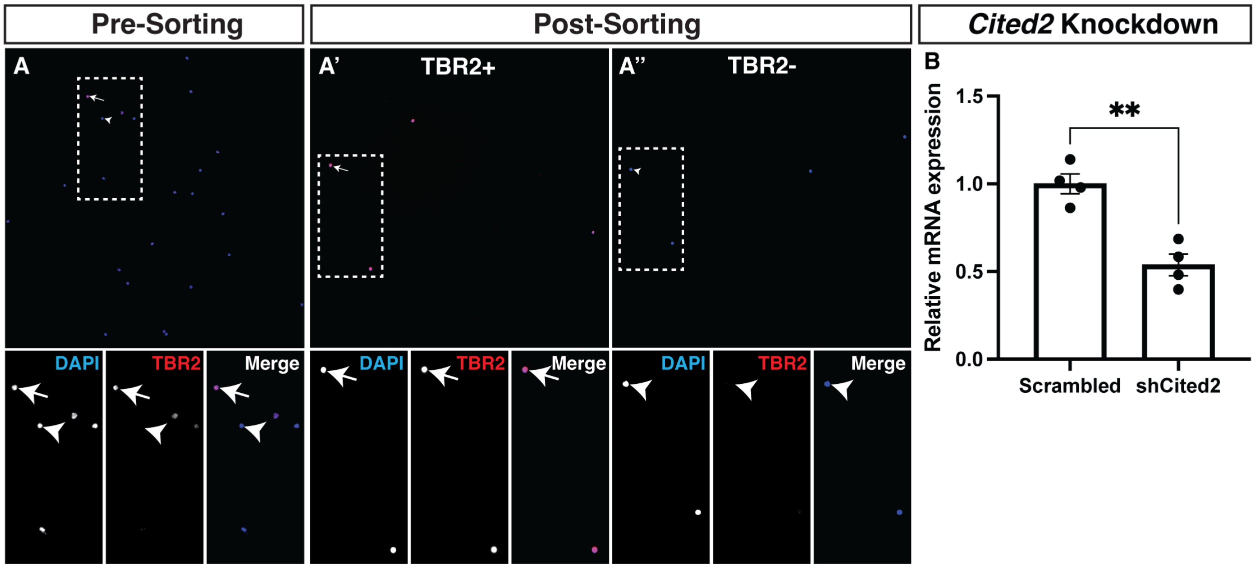

Supplementary Figure 1. Evidence of successful TBR2+ cell sorting and sh*Cited2* knockdown. (A-A'') Successful TBR2+ IPC sorting was validated using immunocytochemistry. (**A**) Pre-sorted single cell suspensions of E15.5 neocortices contain TBR2+ IPCs (TBR2+ DAPI+; white arrow) and other neocortical cell types (TBR2- DAPI+; white arrow heads). IPCs were successfully isolated from other cell types using FACS. (**A'**) All the observed IPCs sorted for the presence of TBR2 express TBR2 (white arrows) and (**A''**) all the observed TBR2- sorted cells were negative for TBR2 expression (white arrow heads). (**B**) Short hairpin mediated *Cited2* knockdown was verified using qPCR. Nucleofection of sh*Cited2* significantly reduced *Cited2* expression relative to scrambled control. n = 4 independent cultures; two-tailed t-test. $p^{**} \leq 0.01$ Error bars denote SEM.
